# Atypical Brain Connectivity During Pragmatic and Semantic Language Processing in Children with Autism

**DOI:** 10.3390/brainsci14111066

**Published:** 2024-10-26

**Authors:** Amparo V. Márquez-García, Vasily A. Vakorin, Nataliia Kozhemiako, Grace Iarocci, Sylvain Moreno, Sam M. Doesburg

**Affiliations:** 1 Department of Biomedical Physiology and Kinesiology, Simon Fraser University, Burnaby, BC V5A 1S6, Canada; amarquez@sfu.ca (A.V.M.-G.); vasily_vakorin@sfu.ca (V.A.V.); 2 Department of Psychiatry, Brigham and Women’s Hospital, Harvard Medical School, Boston, MA 02115, USA; nataliia_kozhemiako@sfu.ca; 3 Department of Psychology, Simon Fraser University, Burnaby, BC V5A 1S6, Canada; grace_iarocci@sfu.ca; 4 Department of School of Interactive Arts & Technology, Simon Fraser University, Burnaby, BC V5A 1S6, Canada; sylvain_moreno@sfu.ca; 5 Institute of Neuroscience and Neurotechnology, Simon Fraser University, Burnaby, BC V5A 1S6, Canada

**Keywords:** autism, fMRI, functional connectivity, pragmatic language, semantic, language, social cognitive neuroscience, idiosyncrasy

## Abstract

Background/Objectives: Children with Autism Spectrum Disorder (ASD) face challenges in social communication due to difficulties in considering context, processing information, and interpreting social cues. This study aims to explore the neural processes related to pragmatic language communication in children with ASD and address the research question of how functional brain connectivity operates during complex pragmatic language tasks. Methods: We examined differences in brain functional connectivity between children with ASD and typically developing peers while they engaged in video recordings of spoken language tasks. We focused on two types of speech acts: semantic and pragmatic. Results: Our results showed differences between groups during the pragmatic and semantic language processing, indicating more idiosyncratic connectivity in children with ASD in the Left Somatomotor and Left Limbic networks, suggesting that these networks play a role in task-dependent functional connectivity. Additionally, these functional differences were mainly localized to the left hemisphere.

## 1. Introduction

Every day, we engage in a continuous exchange of social information, transmitted not only through spoken words but also through nonverbal cues that convey nuanced meanings and emotions. The inability to decipher these contextual cues can lead to confusion and uncertainty regarding the true intent behind received messages. For individuals on the autism spectrum, this struggle is a constant, often overwhelming reality. Many individuals with ASD face significant challenges in navigating conversations due to difficulties in accurately interpreting nonverbal cues and contextual language. This communication barrier leaves them feeling disoriented by their inability to fully grasp the intricacies of interpersonal interactions and the world around them.

ASD is a developmental disability in which there is diminished social communication and restricted/repetitive interests or behaviors [1]. The prevalence of ASD has increased significantly throughout recent decades, with the current overall estimated prevalence of 1 in 36 children, according to the National Center for Health Statistics and Centers for Disease Control and Prevention [2]. ASD is characterized, among other symptoms, by difficulty integrating and understanding inferences appropriately, attributing meaning to different contexts, and understanding language in social interaction [3,4]. Research indicates that challenges in cognitive flexibility contribute to difficulties in considering communication context in individuals with ASD [5]. Furthermore, it has been proposed that the pragmatic language communication impairments observed in ASD involving the interpretation of language within context [6,7,8,9] are associated with a reduced capacity to grasp quickly evolving socially significant cues [10].

Neuroimaging studies comparing brain activity in individuals with ASD to their typically developing (TD) peers have significantly advanced our understanding of the differences between these groups [11,12,13,14,15,16]. Investigating brain connectivity has elucidated the neural mechanisms underlying the atypical cognitive processes observed in individuals with ASD, highlighting how altered connectivity patterns may influence the unique characteristics of ASD behaviors and cognition [17,18]. Symptoms of ASD have been linked to atypical communication patterns within various brain networks, including the Default Mode network and the Salience network [19,20]. Several influential theories further shed light on the neural mechanisms of ASD, particularly the under-connectivity theory, which posits that individuals with ASD often exhibit reduced neural connections within and between key brain regions responsible for language and social processing [12,13]. Variations in connectivity, whether characterized by hyper-connectivity or under-connectivity, correlate closely with the severity of autistic symptoms, showcasing the diverse neural profiles present in individuals with ASD. 

However, studies examining language processing in individuals with ASD compared to TD individuals have yielded inconsistent and often contradictory findings, revealing either increases or decreases in functional connectivity in ASD, or even both [21,22]. Recent research indicates that rather than a consistent pattern of atypical connectivity, a defining characteristic of the ASD brain may be variability in functional connectivity among individuals, often referred to as idiosyncrasy [23]. Idiosyncratic connectivity in ASD reflects irregular patterns of functional connectivity, marked by greater variability in connectivity strengths across different brain networks. This variability can influence the ability to process semantic and pragmatic language, suggesting a unique neural adaptability that distinguishes individuals with ASD from their typically developing peers. Additionally, recent findings indicate that this idiosyncrasy in functional network connectivity may relate to the distinct symptoms commonly associated with ASD [24]. 

Given the increasing evidence of the importance of altered connectivity among regions in ASD, it may be the case that social and pragmatic language difficulties arise from altered idiosyncratic connectivity within and between functional brain networks. Despite the increased knowledge regarding neural activity associated with various aspects of social and pragmatic communication processing, little is known about the brain function of people with ASD when directly observing pragmatically challenging communication situations.

For a more thorough comprehension of pragmatic communication processing, it is crucial to utilize activities that closely mirror real-world interactions. Such tasks reveal the root causes of communication challenges experienced by individuals with ASD in their daily social encounters more effectively than static images or brief phrases. Speech act stimuli are what better represents social communication hurdles and offers a more accurate representation of these challenges. Incorporating video stimuli in research not only minimizes head movements but also lowers arousal levels during functional magnetic resonance imaging (fMRI) scans [25]. 

The current study employs fMRI and video recordings of both pragmatic and semantic speech acts to examine task-dependent network connectivity in children with ASD and their typically developing peers during the processing of complex communication situations. We utilized two sets of nine video clips, totaling eighteen clips, which were organized into two separate runs. Half of the videos contained a semantic component, while the other half focused on a pragmatic component. Additional details regarding the stimulus procedures can be found in Márquez-García et al. (2023) [26].

Social communication difficulties are a hallmark of ASD, and we expect to find evidence of neural correlates of these difficulties. It is essential to clarify that although we utilize the same dataset as Márquez-García et al. (2023) [26], our analysis is distinct, focusing on the architecture of brain connectivity rather than its relationship to cognitive metrics. Therefore, the first aim of this study is to investigate the brain connectivity response of participants to the speech acts videos representing differences between the groups. The second aim of this study was to test each network connectivity independently to identify which networks are driving the differences between groups in the processing of the two separate categories of speech acts (pragmatic and semantic components). 

## 2. Materials and Methods

### 2.1. Participants

Two groups of children participated in this study: children with ASD and TD children. The ASD group included 16 children between 7 and 12 years old (3 females, mean age 10.15 ± 1.32 years old) who were diagnosed with ASD. A diagnosis of ASD in the province of British Columbia, Canada, involves a standardized procedure using the Autism Diagnostic Observation Schedule (ADOS) and the Autism Diagnostic Interview-Revised (ADI-R) conducted by a trained clinician. The children in this study were diagnosed by external clinicians who were not affiliated with the research team. We obtained documentation from parents to confirm that each child met the diagnostic criteria for ASD in the form of a clinical diagnostic report or government funding eligibility report. 

The TD group included 19 children who did not have ASD or other neurodevelopmental disorders (6 females, mean age of 9.55 ± 1.56, age range = 7–12 years old). There were no significant differences in the age groups, and all the participants were right-handed. We excluded participants who did not have normal or corrected-to-normal vision. Participants with major neurological or neuropsychiatric impairments (i.e., Down Syndrome, Tuberous Sclerosis Complex, Epilepsy) other than ASD were excluded. In addition, participants were excluded from this study if they had standard contraindications for MRI scanning procedures, such as metallic implants.

Before MRI scanning, the cognitive abilities of the participants were assessed. None of the participants had intellectual disabilities (IQ < 70). Based on the Full-Scale Intelligence Quotient 2 (FISQ 2) in WASI (subsets: vocabulary and matrix reasoning), there was no statistically significant difference between the groups (ASD: M = 102.75 ± 19.81; TD: M = 110.84 ± 11.44; *p* = 0.16). The participants’ parents also completed the 50-item Autism Quotient (AQ) questionnaire [27], which showed a significant difference between the groups (ASD: M = 32.63 ± 7.83; TD: M = 13 ± 6.94; *p* = 0.001), confirming that individuals in the ASD group had more autistic traits.

This study, a part of a larger multidisciplinary research, was conducted with the utmost respect for ethical standards. Written informed consent was obtained from all participants, and the research was approved by the Simon Fraser University Research Ethics Board. All procedures performed in this study were in strict accordance with the 1964 Helsinki Declaration and its later amendments or comparable ethical standards, ensuring the ethical integrity of this study.

### 2.2. Stimulus Protocol 

**Figure 1:**
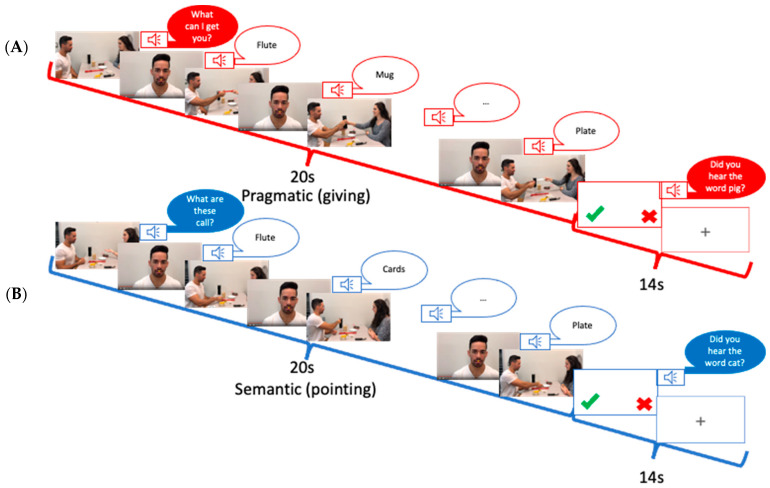
A schematic illustration of the fMRI experiment for the (**A**) semantic condition and (**B**) pragmatic condition. A trial sequence started with a display of objects and communicating actors. A context sentence, e.g., “What are these called?” in the semantic condition (**A**), or “What can I get you?” in the pragmatic condition (**B**), was uttered by the Speaker. Following this, a series of 5 scenes was shown, in which the Speaker’s face appeared together with the critical spoken utterance which served for naming (semantic) vs. requesting (pragmatic). The words were identical for both speech acts. The word scenes were followed by a series of 5 acting scenes, involving the objects mentioned in the worked utterances (handing over an object in the requesting condition or pointing at it in the naming condition).

The stimuli used in the fMRI consisted of two sets of nine video clips, totaling eighteen clips, which were divided into two separate runs. Half of the videos featured a semantic component, while the other half focused on a pragmatic component. Each video depicted two individuals, referred to as the “Partner” and the “Speaker”, seated across from each other at a table. Eight objects were placed on the table between them (Figure 1). The sequence of each video commenced with the Partner saying a context sentence, such as “What are these called?” for the semantic condition or “What can I get you?” for the pragmatic condition. This context determined the speech act in which the Speaker would utter the critical word. Following the context sentence, the Speaker named six of the eight objects on the table. Depending on the context, the critical word was classified as either a Naming speech act (associated with semantic processing) or a Request speech act (linked to pragmatic processing) [28,29]. Each video had a duration of 20 s, and the actions were filmed with one male and one female speaker, who alternated roles in half of the videos.

To ensure consistency, the left–right positions of the characters were counterbalanced, with each position utilized in an equal number of videos. During all scenes, the right hand was used by both speakers to point to or grab the objects. The videos were edited using Apple’s iMovie software, 10.1.13 and the entire sequence of video blocks was presented through Presentation Software 18.2, ensuring a uniform viewing experience. Participants’ attention was assessed at the end of each video block by presenting a critical word, prompting them to confirm whether they had heard that word during the viewing. This measure aimed to keep participants engaged and ensure that they were actively processing the language presented in the videos. 

### 2.3. MRI Data Acquisition

The experimental procedure included functional and anatomical MRI scans. MRI data were recorded at ImageTech, which is Simon Fraser University (SFU)’s imaging facility embedded into a public hospital in the Greater Vancouver area. ImageTech houses a research-dedicated 3T MRI and psychometric assessment facilities. We provided children with snacks, juice, and/or small gifts (toys or movie passes) to maintain concentration and motivation. We obtained assent from the children and informed consent from their parents. We collected high-resolution T1-weighted (T1w) sagittal 3D MPRAGE images covering the whole brain. Five minutes of eyes-open resting-state functional MRI were acquired with a blood-oxygen-level-dependent (BOLD), single-shot, gradient-recalled echo planar imaging (EPI) multi-band sequence. During the recording, the participants were instructed to fixate on a centrally displayed stimulus presented through the MRI-compatible screen. After this, the pragmatic language task used in this research was presented.

MRI scans were acquired with a 3 Tesla Philips Ingenia CX MRI scanner with a 32—channel dStream head coil. fMRI images were acquired using an echo planar imaging GRE-EPI sequence. The acquisition parameters were as follows: TR = 2000 ms, TE = 30 ms, and flip angle = 90°. Data were collected with an in-plane resolution of 3 × 3 mm and slice thickness of 3 mm with matrix dimensions of 80 × 80 voxels and 36 slices. High-resolution T1w images were collected at each session for co-registration of functional images. The acquisition parameters were as follows: TE = 3.7 ms, and flip angle = 8°, FOV = 256 × 242. Slice thickness was 1 mm with matrix dimensions of 256 × 242 voxels and 213 slices, with a sagittal orientation.

### 2.4. Functional MRI Pre-Processing

To pre-process MRI data, we applied the fMRIPrep pipeline, which uses a combination of commonly applied packages such as FSL, ANTs, FreeSurfer, and AFNI [30,31,32,33,34,35,36,37,38,39,40,41]. This pipeline has been designed to provide automatic workflow for pre-processing fMRI data and co-register it with the Montreal Neurological Institute (MNI) brain space. The fMRIPrep pipeline performs several standard pre-processing steps (co-registration, normalization, unwarping, noise component extraction, segmentation, and skull stripping). Refer to our earlier publication [26] for details on the pre-processing pipeline.

### 2.5. Functional Brain Connectivity Analysis 

Each participant’s pre-processed fMRI was further characterized by functional brain connectivity estimated in a network of the cerebral cortex parceled into a relatively small number (compared to the number of voxels) of brain regions (regions of interest or ROIs). The parcellation was defined by the Schaefer atlas [42] at the resolution of 500 ROIs. Each ROI in the parcellation was matched to one of 17 resting-state networks defined by previous fMRI studies [43]. These networks were defined separately for the right and left hemispheres. Thus, there were 34 networks in total. The Schaefer atlas [42] provides a common space where we can link our results to previous findings, thereby facilitating their interpretation. For each ROI, we extracted its characteristic time series by computing the mean fMRI time series averaged across voxels within a given ROI from the Schaefer parcellation. This procedure was performed separately for each run. Then, for each pair of ROIs in the parcellation, we computed three estimates of coordinated activity (functional connectivity), separately for each condition: baseline, semantic and pragmatic. We estimated functional connectivity, using the same number of time points across conditions. More specifically, in run 1, the baseline, semantic and pragmatic conditions had 66, 55, and 55 data points, respectively. In run 2, these numbers were 54, 44, and 44, respectively. Different durations of time series might introduce bias when comparing the estimates of functional connectivity across conditions. To account for this bias, for each run, condition, and each pair of ROIs, we randomly selected 30 data points and then computed distance correlation as a measure of connectivity between two time series. We repeated this procedure 100 times. We then combined all the estimates of distance correlation across both runs and computed its median. The median value was used as an estimate of functional brain connectivity in a given condition for a given pair of brain regions. We then compared the functional connectivity across groups and conditions. 

### 2.6. Statistical Analysis of Differences in Functional Brain Connectivity Across Groups and Conditions

We applied Partial Least Squares (PLS) analysis to statistically assess group and condition differences in functional connectivity. This was performed in a multivariate manner across inter-network or intra-network connections. PLS is a multivariate technique, which decomposes the entire data matrix (participants by fMRI features) into a set of latent variables (LVs), similar to Principal Component Analysis [44,45]. We applied the mean-centered PLS as it is known in the neuroimaging literature and well suited for this type of analysis [46]. The data for PLS analysis were organized as participants within conditions within groups times features (estimates of functional connectivity). In our case, the feature space was defined by all the connections linking two resting-state networks or all the connections within a specific network. Each feature represented the strength of functional connectivity between two ROIs. Note that we used brain parcellation with 500 ROIs, each assigned to 1 of 34 resting-state networks. We thus had 34 × 33/2 = 561 unique pairs of networks, representing inter-network connectivity. Connections linking ROIs within 1 of 34 networks represented intra-network connectivity. In total, we ran 561 + 34 = 595 PLS analyses for an fMRI metric of our choice (e.g., functional connectivity estimated as distance correlation between two time series in the pragmatic or semantic condition). We applied two series of mean-centered PLS analyses, testing two metrics of fMRI connectivity. 

#### 2.6.1. Group Differences Across Both Pragmatic and Semantic Conditions

First, we tested differences in the connectivity across two groups (ASD and TD) and two conditions (semantic and pragmatic). For each subject, the correlation values in both conditions were normalized with respect to the baseline condition. Specifically, before PLS, for each unique pair of ROIs, we subtracted the baseline correlation value from the corresponding correlation values in the semantic and pragmatic conditions. We then performed 595 PLS analyses for all inter-network and intra-network clusters. For each PLS, we identified the latent variable (LV) with the highest explained variance, its overall data-driven contrast across the groups and conditions (a four-dimensional vector), the corresponding *p*-value, and the corresponding set of z-scores, each associated with one ROI pairing. For each PLS analysis, we computed the median values across all the z-scores for a given LV. If the z-score median was negative, we multiplied the overall contrast and z-scores by −1. This procedure ensured that the z-scores were always skewed towards positive values, which facilitated the comparison of overall contrasts across PLS analyses. *p*-values from all PLS analyses were then corrected for multiple comparison under the framework of false discovery rate (FDR) based on the Benjamani–Hochberg procedure [47]. In Section 3, we show the network pairings at two levels of significance: 5% and 10% of FDR. In essence, these results demonstrated differences in functional connectivity between ASD and TD across two conditions.

#### 2.6.2. Group Differences for Pragmatic and Semantic Conditions

In the second series of mean-centered PLS analyses, we aimed to investigate to what degree differences in the connectivity between the semantic and pragmatic conditions differed across the experimental groups ASD and TD (differences-in-differences). For each subject, for each ROI pairing, we computed the absolute value of the difference between the corresponding correlation values in the semantic and pragmatic conditions. Taking the absolute value implied that we ignored the directionality of the effect, regardless of the sign of changes in the correlation value. In other words, positive or negative changes (semantic > pragmatic or pragmatic > semantic) were equalized. Such an approach reflects the concept of idiosyncrasy in ASD, wherein only deviations from the control group are considered, regardless of the directionality of the effects [48]. We then performed another series of 595 PLS analyses for all inter-network and intra-network clusters. As before, for each PLS, we identified the latent variable LV with the highest explained variance, its overall data-driven contrast across the groups (a two-dimensional vector; only one condition, namely, a difference between the semantic and pragmatic connectivity), its *p*-value, and corresponding z-scores, each associated with one connection linking two ROIs. The *p*-values were corrected for multiple comparison using the same Benjamani–Hochberg procedure [47]. We reported the results at two levels of significance: 5% and 10% of FDR.

## 3. Results

### 3.1. Idiosyncratic Network Connectivity During Pragmatic Language Processing 

**Figure 2:**
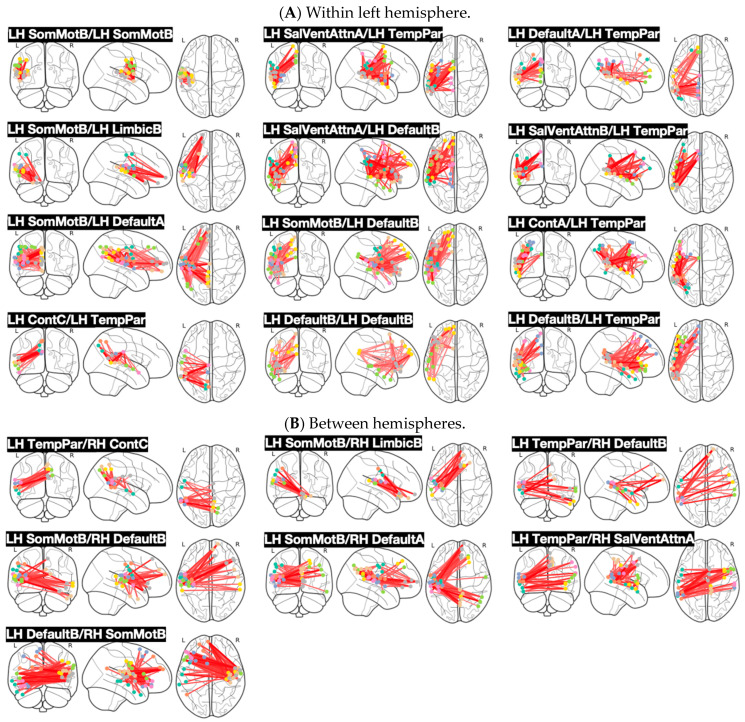
Connectivity graphs for individual network pairings that were found to be significant in expressing group differences. (**A**) Significant connections within the left hemisphere. (**B**) Significant connections between hemispheres. The red lines represent each individual connection between two ROIs belonging to two different networks. The dots represent different ROIs. Every connection contributed to the overall data-driven contrast representing differences between ASD and TD. Each connection was associated with a z-score and all the z-scores distributions were positively skewed, indicating increased task-dependent functional connectivity in children with ASD. In this figure, we show connections higher than 2.5.

We considered 17 fMRI networks per hemisphere, which were represented by 500 brain ROIs according to the Schaefer Atlas [42]. We investigated the differences in functional brain connectivity separately for each pair of networks (561 pairs in total for inter-network connectivity) and for each of 34 networks (intra-network connectivity). We identified a set of network pairings, expressing significant differences across groups and conditions at two levels of significance (5% and 10% of FDR). To see the maps of z-scores, refer to Appendix A. Figure 2 shows the individual connections linking individual ROIs within the functional networks. Specifically, we provided a graph of connections for each significant network pairing, which robustly contributed to the contrast representing differences between ASD and TD. Note that each connection was associated with a z-score. By design, all the distributions of z-scores (for all network pairings) were positively skewed. For each pair of the functional networks, we applied a threshold for the z-scores and illustrated only those connections with a z-score higher than 2.5. 

**Figure 3:**
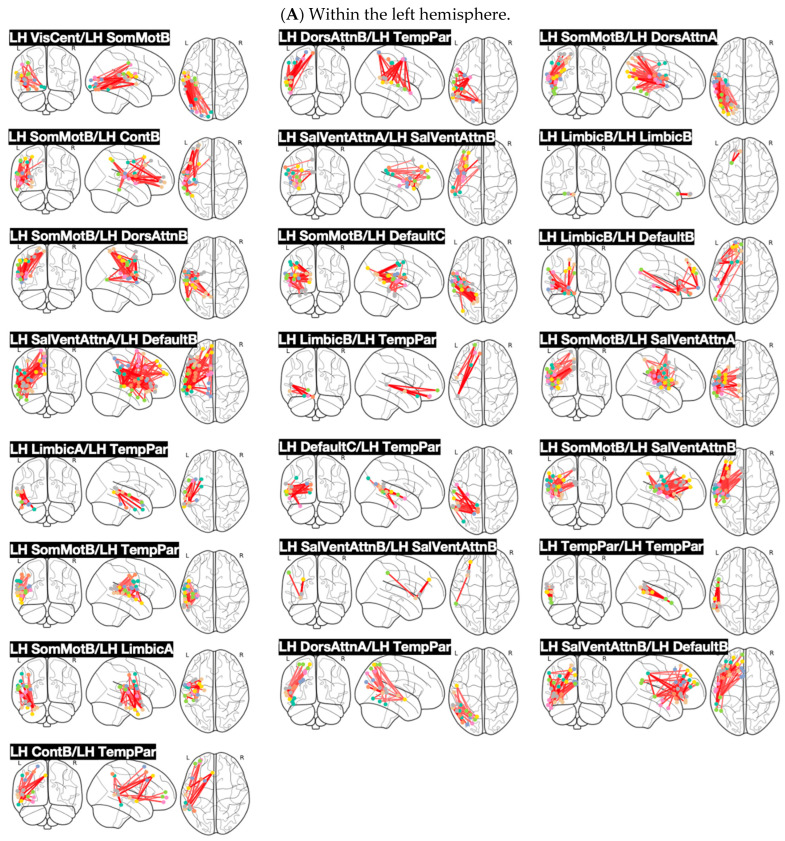

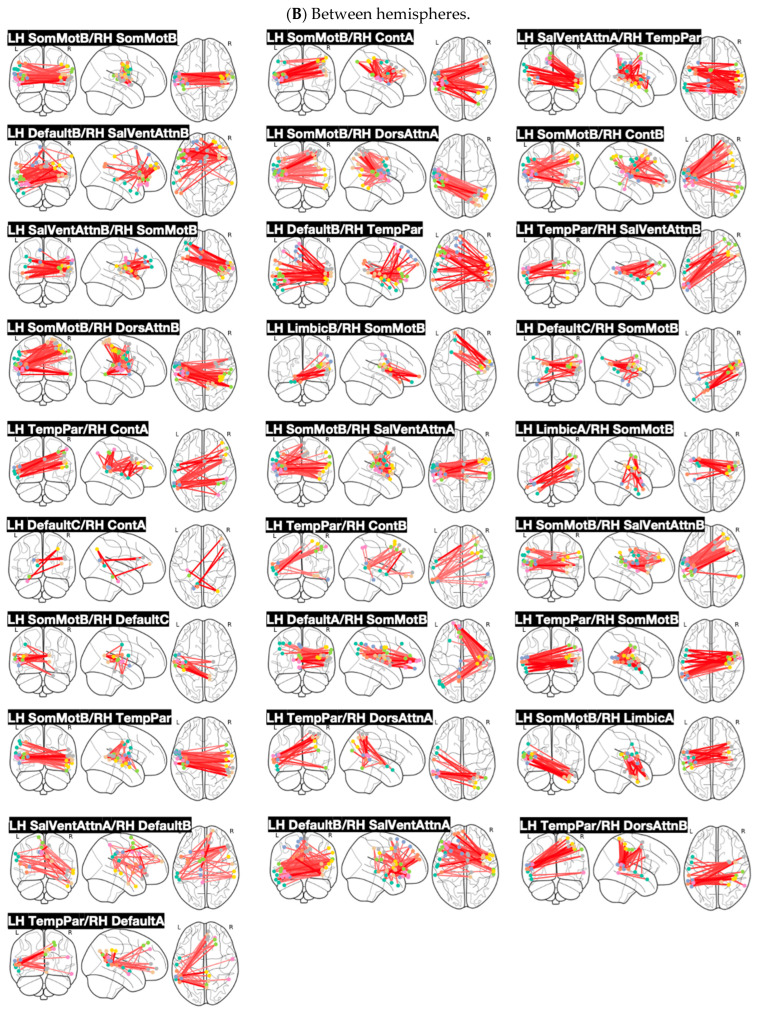

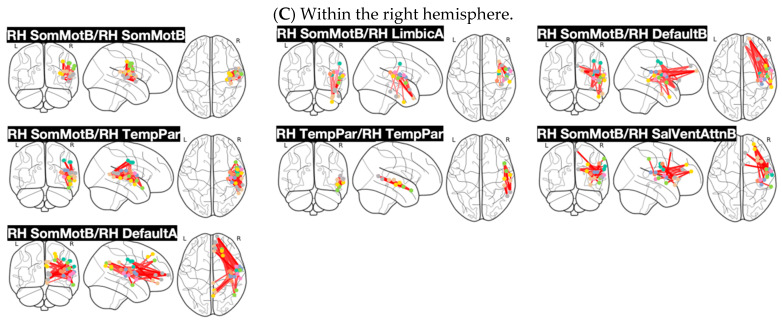
Brains showing each network separately and the connections that showed statistical significance at 10%. (**A**) Significant connections within the left hemisphere. (**B**) Significant connections between hemispheres. (**C**) Significant connections within the right hemisphere. The red lines represent each network pairing marked as significant in the analysis. The dots represent different ROIs. Every connection contributed to the overall data-driven contrast representing differences between ASD and TD. Each connection was associated with a z-score and all the z-score distributions were positively skewed, indicating increased task-dependent functional connectivity in children with ASD.

Figure 2 shows the connectivity graphs for the network pairings, which were found significant at the 5% FDR. Figure 3 shows the connectivity graphs for the network pairings, which were significant at the 10% FDR but not shown in Figure 2. More specifically, Figure 2A shows the connections within the left hemisphere, whereas Figure 2B shows inter-hemispheric connections. In turn, Figure 3A,B illustrate the connectivity graphs representing interactions within the left and right hemispheres, respectively. Figure 2B represents the interactions across the hemispheres. The robust group differences have been supported by the following network interactions: left hemisphere Somatomotor B (SomMotB), left hemisphere Salience/ventral Attention A (SalVentAttnA), left hemisphere Salience/ventral attention B (SalVentAttnB), left hemisphere Limbic B (LimbicB), left hemisphere Control A (ContA), left hemisphere Control B (ContB), left hemisphere Default-Mode A (DefaultA), left hemisphere Default-Mode B (DefaultB), left hemisphere Temporo Parietal (TempPar), right hemisphere Somatomotor B (SomMotB), right hemisphere Salience/Ventral Attention A (SalVentAttnA), right hemisphere Limbic B (LimbicB), right hemisphere Control C (ContC), right hemisphere Default-Mode A (DefaultA), right hemisphere Default-Mode B (DefaultB).

Figure 3 individually shows each pair of networks that represent statistical significance at 10% of FDR (excluding the ones at 5% significance of FDR). Figure 3A shows connections within the left hemisphere, Figure 3B shows connections between hemispheres, and Figure 3C shows connections within the right hemisphere.

### 3.2. Idiosyncratic Network Connectivity During Pragmatic Language Processing in ASD

**Figure 4:**
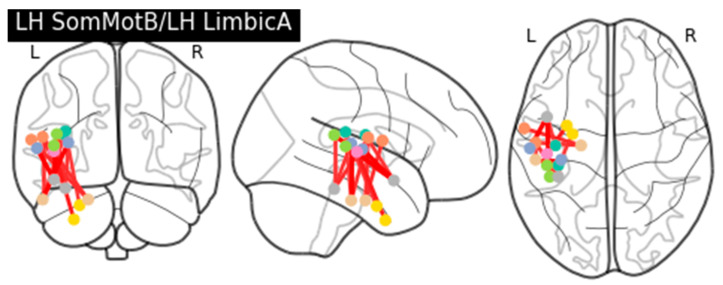
Brains showing each network separately form the pair of networks associated with significant increases in idiosyncratic task dependent connectivity in children with ASD.

We also tested the absolute differences in functional connectivity between the semantic and pragmatic task conditions across the groups. The analysis was performed similarly to how we tested functional connectivity across the two groups and two conditions. Only in this case was the contrast two-dimensional, which represented the overall differences between ASD and TD. We explored differences in functional brain connectivity between the pragmatic and semantic conditions across the groups separately for each pair of networks (again, 561 network pairings in total) and for each of the 34 networks. The *p*-values were corrected for multiple comparisons with FDR. At the level of 5% or 10% FDR, we identified one network pairing, specifically, the Somatomotor B (SomMotB) and Limbic A (LimbicA) networks, both located in the left hemisphere. The distribution of z-scores, each associated with two ROIs linking these two networks, was positively skewed towards the positive values. Together with the overall contrast defined as ASD > TD, this indicates that the idiosyncrasy in the functional connectivity between the pragmatic and semantic task processing was higher in the ASD group compared to the TD group. In other words, these connections showed a greater increase in idiosyncratic functional connectivity for the ASD children, relative to the TD children, when contrasting pragmatic and semantic task conditions. Conceptually, this is similar to a group-by-condition interaction reflecting increased idiosyncrasy specific to pragmatic language processing. Figure 4 shows the connectivity graph, which includes connections with the z-scores higher than 2.5. 

## 4. Discussion

The present study investigated differences in brain connectivity between children with ASD and their TD peers while they processed two categories of speech acts: semantic (naming) and pragmatic (giving). We analyzed all the networks identified in the Schaefer brain atlas and discovered that the left Somatomotor and left Limbic networks were particularly associated with the differences observed between the groups in processing these speech acts. Additionally, we found that the ASD group exhibited higher levels of idiosyncrasy compared to the TD group.

Higher idiosyncrasy in individuals with ASD has been previously documented and is linked to the characteristic symptomatology of the disorder [23,24]. Our findings extend this understanding by demonstrating that higher idiosyncrasy is also present in relation to pragmatic language difficulties. This unique neural connectivity may affect the integration of sensory information, language processing, and social cognition, contributing to pragmatic language deficits and challenges in social communication [49]. As highlighted by the pioneering research of Pegado et al. in 2020 [50], individuals with ASD often display idiosyncratic neural connectivity in higher-level perceptual areas within the visual and auditory domains. This suggests that such idiosyncrasies may arise from the cumulative impact of sensory processing on higher-order cognition, influencing how individuals with ASD perceive and respond to social cues during communication, an essential insight for understanding the condition.

Moreover, the networks exhibiting group differences in task-dependent connectivity for language processing across both semantic and pragmatic conditions included the Limbic, Somatomotor, Salience/Ventral Attention, Default-Mode, and Control networks. These networks displayed a similar trend, with increased idiosyncrasy observed in the ASD group compared to their TD counterparts. Previous studies have reported similar trends in various tasks, such as emotional face processing [51] and resting-state connectivity [52,53]. Notably, the differences between groups were predominantly lateralized in the left hemisphere, consistent with prior research highlighting atypical functional lateralization of language in ASD [54,55,56,57,58,59,60].

The increased idiosyncratic connectivity observed in children with ASD could indicate that individualized therapies tailored to the unique neural profiles of each child might be beneficial. This approach aligns with the emerging trend in personalized medicine, where interventions are customized based on individual neurological and behavioral characteristics.

### 4.1. Limbic Network

In our study, increased idiosyncratic connectivity was observed in children with ASD while processing the language task (including both conditions), particularly in connections involving the Limbic network and other networks. This includes the left hemisphere Limbic network B and the right hemisphere Limbic network B, both associated with the anterior Limbic network, which encompasses the hypothalamus and an anterior group of thalamic nuclei. Additionally, differences between groups in processing the two conditions (pragmatic and semantic, separately) indicated a more idiosyncratic connectivity in children with ASD between the left Somatomotor network and the left Limbic network. These results align with previous studies that have noted atypical functions and structures within the Frontotemporal and Limbic networks, which are connected to social and pragmatic language difficulties [61,62,63].

The literature consistently indicates that the Limbic system plays a crucial role in social and emotional functioning [64], with established differences in Limbic function and connectivity observed in individuals with ASD [65]. Notably, structures such as the cingulate gyrus and amygdala have been implicated in specific facets of social cognition, mediating cognitive and affective processes that are atypical in ASD. Differences in the basolateral circuit, a Limbic structure integral to social cognition, have also been reported in individuals with ASD [66]. In a systematic review conducted by Caroline Larson in 2022 [67], the functional connectivity of language networks in individuals with ASD was examined through a review of 96 studies utilizing fMRI. The review highlights the critical role of the Limbic system, particularly the cingulate cortex and amygdala, in emotion regulation, social behavior, and memory—all essential for language processing and social interaction. Furthermore, findings indicate that individuals with ASD may exhibit local over-connectivity within certain Limbic-related areas, such as the cingulate gyrus, while experiencing global under-connectivity with other brain regions, including the prefrontal cortex and temporal lobe, which impacts language integration and social cognition. The review also notes correlations between Limbic connectivity and ASD symptomatology, suggesting that under-connectivity may be associated with social communication difficulties.

Supporting these findings, a study conducted by Rui Zhou and colleagues in 2024 [68] investigated the connectivity patterns of the Limbic network in individuals with ASD using resting-state functional magnetic resonance imaging (rs-fMRI). The study revealed increased inter-network connectivity between the Limbic network and the Subcortical network, indicating potential compensatory hyper-connectivity related to emotional processing. Conversely, the researchers noted a significant decrease in inter-network connectivity between the Limbic network and the Default Mode network (DMN), suggesting a disconnect that may impact self-referential thought processes and social communication abilities. In conclusion, individuals with ASD demonstrate distinct differences from TD individuals in language processing and the functioning of social and emotional processing areas, such as the Limbic network, especially while observing speech acts in videos. Overall, these findings emphasize the importance of the Limbic network in understanding the neurological basis of ASD and suggest avenues for enhancing diagnostic and therapeutic strategies.

### 4.2. Somatomotor Network

In our study, the Somatomotor network, also known as the Sensorimotor network, exhibited significant differences between groups in connectivity within and between different networks. The Somatomotor network encompasses both somatosensory regions in the postcentral gyrus and motor regions in the precentral gyrus, extending into the supplementary motor areas [69]. Functional connectivity involving the Sensorimotor network has been implicated in the development of motor skills as well as the core symptoms of ASD, which include social difficulties and restricted and repetitive behaviors, as demonstrated by prospective studies involving infant siblings. More specifically, research has shown that functional connectivity within the motor network and the DMN correlates with milestones such as the onset of walking and gross motor functioning [70]. 

Further insights are provided by the work of Rui Zhou et al. (2024) [68], which examines altered connectivity patterns in individuals with ASD. Their findings indicate a significant decrease in inter-network connectivity involving the Somatomotor network, particularly with the Salience/Ventral Attention network (SVAN) and the Frontoparietal network. This reduction suggests a functional segregation or altered processing between sensory and motor areas and cognitive networks. Conversely, this study points to an increase in inter-network connectivity between certain regions of the Limbic network and the Somatomotor network, suggesting a potentially heightened interaction between these networks.

Moreover, A.S. Nunes et al. (2019) [24] investigated the idiosyncratic organization of cortical networks in individuals with ASD using rs-fMRI. Their study reveals that individuals with ASD exhibit a more variable organization of intrinsic connectivity networks (ICNs), particularly within the Somatomotor network. This variability is characterized by a significant reduction in the stability of network connectivity in the Somatomotor network among participants with ASD when compared to TD individuals, indicating that the activation patterns of the Somatomotor network may be less consistent. Furthermore, the study finds a negative correlation between the idiosyncratic organization of the Somatomotor network and the severity of ASD symptoms, as measured by the Autism Diagnostic Observation Schedule (ADOS). This correlation suggests that greater variability in Somatomotor network connectivity is associated with more pronounced symptomatology, underscoring the potential role that altered Somatomotor network connectivity may play in the social and sensory processing difficulties commonly observed in ASD.

In conclusion, the examination of the Somatomotor network reveals critical insights into the neurodevelopmental differences present in individuals ASD. The variations in connectivity not only highlight the complexities of motor and sensory integration but also suggest potential pathways for understanding the neurological underpinnings of social challenges in pragmatic language communication. 

### 4.3. Default-Mode Network

In our study, we found distinct differences in the connectivity of the Default Mode network (DMN) between children with ASD and TD children during the proposed language task. The DMN is composed of several key regions, including the posterior cingulate cortex, precuneus, medial prefrontal cortex, temporoparietal junction, and hippocampus [71,72]. 

Extensive evidence suggests that structural and functional disruptions to crucial nodes within the DMN, along with abnormal connectivity patterns with other brain regions, significantly contribute to the symptomatology of ASD [12,73,74,75,76,77,78,79,80,81,82,83]. The current literature indicates that the atypical integration of self-referential and social information within the DMN may underlie the social difficulties commonly observed in individuals with ASD. Our results suggest that this integration also influences language processing, particularly during the interpretation of speech acts in videos.

Furthermore, connectivity between visual processing networks and higher-order networks, such as the Dorsal Attention network (DAN) and posterior DMN, is associated with the initiation of joint attention [84]. Additionally, functional connectivity patterns among visual areas, the DMN, and the Frontoparietal Control network are related to specific aspects of restricted and repetitive behaviors [85].

A recent study by Rui Zhou and colleagues (2024) [68] highlighted reductions in intra-network connectivity within both the DMN and the Dorsal Attention network, suggesting disruptions in attention and self-processing that are prevalent in ASD. Moreover, a meta-analysis by Wang et al. (2021) [86] focusing on resting-state connectivity in the DMN among individuals with ASD found evidence of over-connectivity among visual, motor, and social–emotional regions within the DMN (e.g., right middle temporal gyrus [MTG], right supramarginal gyrus [SMG], cerebellum), alongside under-connectivity primarily linked to the angular gyrus. These results point to a pattern of spatially distant global over-connectivity combined with spatially proximal local under-connectivity.

In contrast, research by Vissers et al. (2012) [87] provided robust evidence of spatially distant global under-connectivity related to the DMN and areas associated with social-emotional processing (e.g., the insula and somatosensory regions), while indicating minimal evidence of spatially proximal local over-connectivity.

### 4.4. Salience/Ventral Attention Network

We observed notable differences between the groups concerning the Salience/Ventral Attention network, specifically within the left hemisphere Salience/Ventral Attention A, left hemisphere Salience/Ventral Attention B, and right hemisphere Salience/Ventral Attention A. This network plays a crucial role in various attentional processes, including error monitoring, selective attention, and task switching [88,89]. Our findings indicate that children with ASD exhibit differences in the strength of functional connectivity within the Salience/Ventral Attention networks compared to their typically developing peers. These results align with a growing body of research highlighting atypical functional connectivity of large-scale executive networks both at rest [90,91,92] and during task performance [23,93,94,95,96].

### 4.5. Implications of Idiosyncratic Connectivity in the Cerebellum and Mirror Neurons in ASD

Idiosyncratic connectivity patterns in the cerebellum significantly contribute to the manifestation of ASD symptoms. Disruptions in the cerebellum’s functional connectivity with brain regions related to social behavior may contribute to the social communication challenges faced by children with ASD [97]. The cerebellum plays a crucial role in ASD by influencing cognitive and affective development. Its disruptions during sensitive periods can impair the maturation of neural circuits related to social behavior, including the cerebellar–thalamic–cortical pathways, fronto-cerebellar connections, and circuits linking the cerebellum to the amygdala and prefrontal cortex. Research indicates that cerebellar dysfunction correlates with autism’s core symptoms, including social impairments. Early-life injuries to the cerebellum significantly increase autism risk, supporting the idea that the cerebellum integrates sensory and emotional information, which is vital for social capacities [98]. 

Recent work has shown that the cerebellum critically modulates mirror neuron system (MNS) activity and impacts the networks related to social cognition. This relationship suggests potential inhibitory mechanisms through which the cerebellum influences both motor and cognitive systems, enhancing our understanding of ASD pathophysiology [99,100]. Notably, cerebellar-specific genetic mutations have been linked to autistic-like behavior [101], emphasizing the genetic complexity underlying cerebellar dysfunction in ASD [102]. Furthermore, cerebellar connectivity with regions such as the right temporo-parietal junction is associated with social abilities and cognitive functions [103]. Atypical connectivity patterns, particularly in the sensorimotor cerebellum, have been linked to sensory over-responsivity in ASD [97]. 

In conclusion, while the manuscript has focused on other networks, recognizing the cerebellum’s role in modulating MNS activity enhances the discussion around the interplay between motor and cognitive systems in pragmatic language deficits in ASD.

### 4.6. Limitations and Future Directions

One limitation of the present study is its relatively small sample size, which was influenced by the challenges of acquiring usable imaging data in this age group. In addition, it is worth considering that the additional fundamental group differences in network connectivity may have been masked by differential maturational trajectories across the sampled age range [23].

In the context of future research directions, we emphasize the importance of longitudinal studies to enhance our understanding of brain connectivity changes over time in children with ASD. Such investigations can provide insights into the dynamic interplay between atypical connectivity patterns and language acquisition, potentially revealing critical periods where interventions may be most effective. Furthermore, it would be beneficial for future research to incorporate age-related covariates in their analyses to statistically parse the specific contributions of age. Such analyses could refine our understanding of how age impacts the neural mechanisms of language processing and connectivity in children with ASD. Moreover, future research should aim for more comprehensive demographic matching to strengthen findings in this area.

Additionally, while our focus was primarily on cortical networks, we recognize the potential significance of the cerebellum in the context of ASD [104]. Moving forward, we suggest that future studies include the cerebellum in their data collection and analyses, exploring its implications based on the emerging literature surrounding its role in social cognition and action observation.

## 5. Conclusions

Our study investigates the neural connectivity involved in social communication and emphasizes the processing of speech acts in children with ASD. We found distinct differences in how children with and without ASD process speech acts related to pragmatic and semantic language, particularly within the Limbic and Somatomotor networks. These areas are crucial for social cognition, emotional processing, and motor skills. Furthermore, we observed idiosyncratic functional connectivity in children with ASD during pragmatic language processing. These findings align with existing evidence indicating that children with ASD exhibit lateralized differences and heightened variability in connectivity compared to typically developing children. 

## Figures and Tables

**Figure A1:**
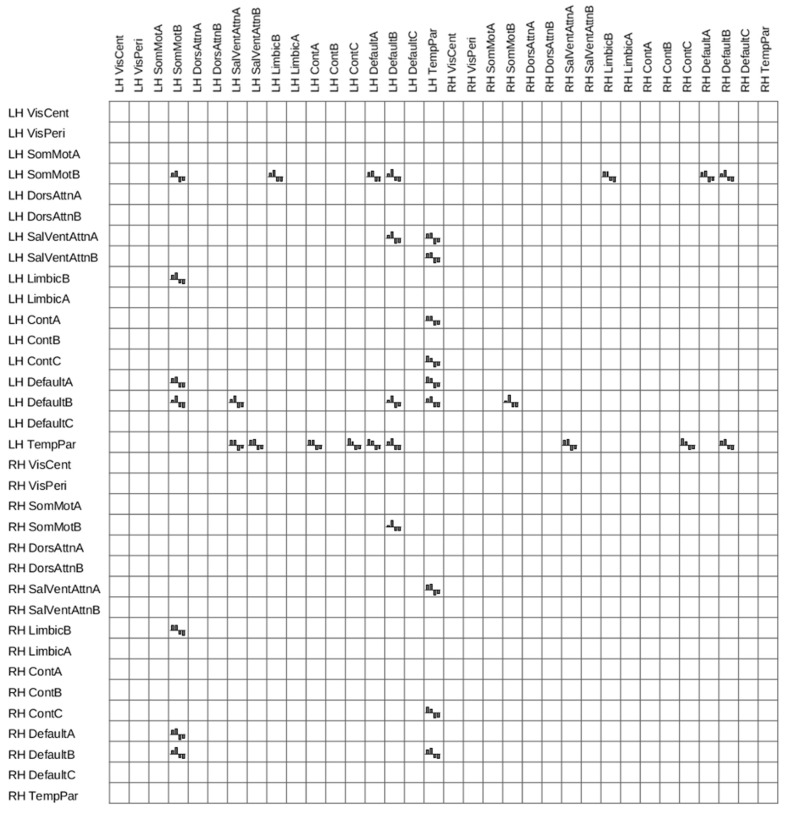
Map of z-scores showing the contrast of ASD vs. TD children, with the semantic and pragmatic conditions tested together. The map of inter-network connectivity presents each data-driven contrast from PLS at 5% significance. Each network presents a four-dim dimensional contrast. The labels, starting from left to right bars, are ASD group, pragmatic condition; ASD group, semantic condition; TD group, pragmatic condition; and TD group, semantic condition.

**Figure A2:**
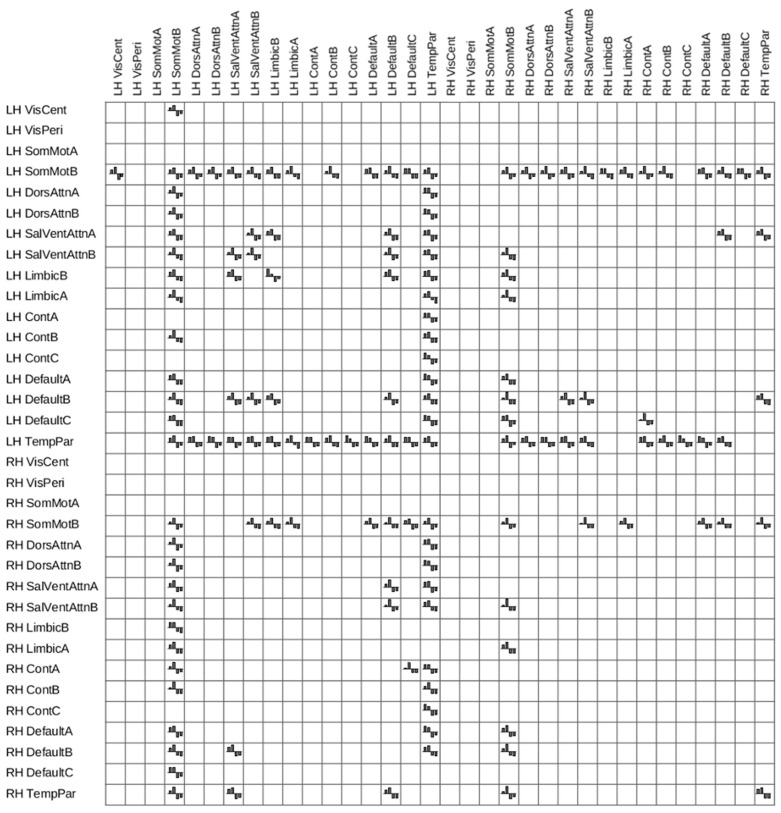
Map of z-scores showing the contrast of ASD vs. TD children, with the semantic and pragmatic conditions tested together. The map of inter-network connectivity presents each data-driven contrast from PLS at a 10% significance threshold. Each network presents a four-dimensional contrast. The labels, starting from left to right bars, are ASD group, pragmatic condition; ASD group, semantic condition; TD group, pragmatic condition; and TD group, semantic condition.

## Data Availability

Data available upon request due to ethical reasons.

## References

[1] American Psychiatric Association (2013). Diagnostic and Statistical Manual of Mental Disorders (DSM-5®). https://books.google.ca/books?hl=en&lr=&id=-JivBAAAQBAJ&oi=fnd&pg=PT15&dq=DSM-5&ots=cfOO35NKtd&sig=Xr2RdL-IOEPN3hgJNrz0byFuJXw#v=onepage&q=DSM-5&f=false.

[2] National Center for Health Statistics (2020). Data & Statistics on Autism Spectrum Disorder. https://www.cdc.gov/nchs/index.htm.

[3] Eguílaz N.C., García J.N. (2011). Dificultades en la percatación rápida de incongruencias en el trastorno de aprendizaje procedimental: Posible disfunción de la coherencia central. Rev. De Neurol..

[4] López B., Leekam S.R. (2007). Teoría de la coherencia central: Una revisión de los supuestos teóricos. J. Study Educ. Dev..

[5] Kissine M. (2012). Pragmatics, cognitive flexibility and autism spectrum disorders. Mind Lang..

[6] Berenguer C., Miranda A., Colomer C., Baixauli I., Roselló B. (2018). Contribution of Theory of Mind, Executive Functioning, and Pragmatics to Socialization Behaviors of Children with High-Functioning Autism. J. Autism Dev. Disord..

[7] Klusek J., Martin G.E., Losh M., Klusek G.E.M.J. (2013). Physiological arousal in autism and fragile X syndrome: Group comparisons and links with pragmatic language. Am. J. Intellect. Dev. Disabil..

[8] Larkin F., Hobson J.A., Hobson R.P., Tolmie A. (2017). Collaborative competence in dialogue: Pragmatic language impairment as a window onto the psychopathology of autism. Res. Autism Spectr. Disord..

[9] Martin G.E., Bush L., Klusek J., Patel S., Losh M. (2018). A multimethod analysis of pragmatic skills in children and adolescents with fragile X syndrome, autism spectrum disorder, and down syndrome. J. Speech Lang. Hear. Res..

[10] Vanmarcke S., van Esch L., Van der Hallen R., Evers K., Noens I., Steyaert J., Wagemans J. (2016). Gist perception in adolescents with and without ASD: Ultra-rapid categorization of meaningful real-life scenes. Res. Autism Spectr. Disord..

[11] Harris G.J., Chabris C.F., Clark J., Urban T., Aharon I., Steele S., McGrath L., Condouris K., Tager-Flusberg H. (2006). Brain activation during semantic processing in autism spectrum disorders via functional magnetic resonance imaging. Brain Cogn..

[12] Just M.A., Cherkassky V.L., Buchweitz A., Keller T.A., Mitchell T.M. (2014). Identifying autism from neural representations of social interactions: Neurocognitive markers of autism. PLoS ONE.

[13] Kana R.K., Keller T.A., Cherkassky V.L., Minshew N.J., Just M.A. (2006). Sentence comprehension in autism: Thinking in pictures with decreased functional connectivity. Brain.

[14] Knaus T.A., Silver A.M., Kennedy M., Lindgren K.A., Kelli C., Siegel J., Tager-flusberg H. (2010). Language laterality in autism spectrum disorder and typical controls: A functional, volumetric, and diffusion tensor MRI study. Brain Lang..

[15] Lawrence K.E., Hernandez L.M., Bookheimer S.Y., Dapretto M. (2019). Atypical longitudinal development of functional connectivity in adolescents with autism spectrum disorder. Autism Res..

[16] Rohr C.S., Kamal S., Bray S. (2020). Building functional connectivity neuromarkers of behavioral self-regulation across children with and without Autism Spectrum Disorder. Dev. Cogn. Neurosci..

[17] Courchesne E., Carper R., Akshoomoff N. (2003). Evidence of Brain Overgrowth in the First Year of Life in Autism. JAMA.

[18] Courchesne E., Redcay E., Kennedy D.P. (2004). The autistic brain: Birth through adulthood. Curr. Opin. Neurol..

[19] Müller R.-A., Shih P., Keehn B., Deyoe J.R., Leyden K.M., Shukla D.K. (2011). Underconnected, but how? A survey of functional connectivity MRI studies in autism spectrum disorders. Cereb. Cortex.

[20] Uddin L.Q., Supekar K., Lynch C.J., Khouzam A., Phillips J., Feinstein C., Ryali S., Menon V. (2013). Salience network–based classification and prediction of symptom severity in children with autism. JAMA Psychiatry.

[21] Hull J.V., Dokovna L.B., Jacokes Z.J., Torgerson C.M., Irimia A., Van Horn J.D. (2016). Resting-State Functional Connectivity in Autism Spectrum Disorders: A Review. Front. Psychiatry.

[22] Rane P., Cochran D., Hodge S.M.M., Haselgrove C., Kennedy D.N., Frazier J.A. (2015). Connectivity in Autism. Harv. Rev. Psychiatry.

[23] Uddin L.Q., Supekar K., Lynch C.J., Cheng K.M., Odriozola P., Barth M.E., Phillips J., Feinstein C., Abrams D.A., Menon V. (2015). Brain State Differentiation and Behavioral Inflexibility in Autism. Cereb. Cortex.

[24] Nunes A., Peatfield N., Vakorin V., Doesburg S.M. (2019). Idiosyncratic organization of cortical networks in autism spectrum disorder. NeuroImage.

[25] Vanderwal T., Kelly C., Eilbott J., Mayes L.C., Castellanos F.X. (2015). Inscapes: A movie paradigm to improve compliance in functional magnetic resonance imaging. NeuroImage.

[26] Márquez-García A.V., Ng B.K., Iarocci G., Moreno S., Vakorin V.A., Doesburg S.M. (2023). Atypical Associations between Functional Connectivity during Pragmatic and Semantic Language Processing and Cognitive Abilities in Children with Autism. Brain Sci..

[27] Baron-Cohen S., Wheelwright S., Skinner R., Martin J., Clubley E. (2001). The autism-spectrum quotient (AQ): Evidence from asperger syndrome/high-functioning autism, males and females, scientists and mathematicians. J. Autism Dev. Disord..

[28] Egorova N., Shtyrov Y., Pulvermüller F. (2013). Early and parallel processing of pragmatic and semantic information in speech acts: Neurophysiological evidence. Front. Hum. Neurosci..

[29] Egorova N., Shtyrov Y., Pulvermüller F. (2016). Brain basis of communicative actions in language. NeuroImage.

[30] Dale A.M., Sereno M.I. (1993). Improved localizadon of cortical activity by combining EEG and MEG with MRI cortical surface reconstruction: A linear approach. J. Cogn. Neurosci..

[31] Dale A.M., Fischl B., Sereno M.I. (1999). Cortical surface-based analysis: I. Segmentation and surface reconstruction. Neuroimage.

[32] Fischl B., Dale A.M. (2000). Measuring the thickness of the human cerebral cortex from magnetic resonance images. Proc. Natl. Acad. Sci. USA.

[33] Fischl B., Liu A., Dale A.M. (2001). Automated manifold surgery: Constructing geometrically accurate and topologically correct models of the human cerebral cortex. IEEE Trans. Med. Imaging.

[34] Fischl B., Salat D.H., Busa E., Albert M., Dieterich M., Haselgrove C., van der Kouwe A., Killiany R., Kennedy D., Dale A.M. (2002). Whole brain segmentation: Automated labeling of neuroanatomical structures in the human brain. Neuron.

[35] Fischl B., Salat D.H., van der Kouwe A.J., Makris N., Ségonne F., Quinn B.T., Dale A.M. (2004). Sequence-independent segmentation of magnetic resonance images. NeuroImage.

[36] Fischl B., Sereno M.I., Tootell R.B., Dale A.M. (1999). High-resolution intersubject averaging and a coordinate system for the cortical surface. Hum. Brain Mapp..

[37] Fischl B., van der Kouwe A., Destrieux C., Halgren E., Ségonne F., Salat D.H., Busa E., Seidman L.J., Goldstein J., Kennedy D. (2004). Automatically parcellating the human cerebral cortex. Cereb. Cortex.

[38] Han X., Jovicich J., Salat D., van der Kouwe A., Quinn B., Czanner S., Busa E., Pacheco J., Albert M., Killiany R. (2006). Reliability of MRI-derived measurements of human cerebral cortical thickness: The effects of field strength, scanner upgrade and manufacturer. NeuroImage.

[39] Jovicich J., Czanner S., Greve D., Haley E., van der Kouwe A., Gollub R., Kennedy D., Schmitt F., Brown G., MacFall J. (2006). Reliability in multi-site structural MRI studies: Effects of gradient non-linearity correction on phantom and human data. NeuroImage.

[40] Reuter M., Rosas H.D., Fischl B. (2010). Highly accurate inverse consistent registration: A robust approach. NeuroImage.

[41] Reuter M., Schmansky N.J., Rosas H.D., Fischl B. (2012). Within-subject template estimation for unbiased longitudinal image analysis. NeuroImage.

[42] Schaefer A., Kong R., Gordon E.M., Laumann T.O., Zuo X.-N., Holmes A.J., Eickhoff S.B., Yeo B.T.T. (2018). Local-Global Parcellation of the Human Cerebral Cortex from Intrinsic Functional Connectivity MRI. Cereb. Cortex.

[43] Thomas Yeo B.T., Krienen F.M., Sepulcre J., Sabuncu M.R., Lashkari D., Hollinshead M., Roffman J.L., Smoller J.W., Zöllei L., Polimeni J.R. (2011). The organization of the human cerebral cortex estimated by intrinsic functional connectivity. J. Neurophysiol..

[44] Lobaugh N.J., West R., McIntosh A.R. (2001). Spatiotemporal analysis of experimental differences in event-related potential data with partial least squares. Psychophysiology.

[45] McIntosh A.R., Lobaugh N.J. (2004). Partial least squares analysis of neuroimaging data: Applications and advances. NeuroImage.

[46] Krishnan A., Williams L.J., McIntosh A.R., Abdi H. (2011). Partial Least Squares (PLS) methods for neuroimaging: A tutorial and review. NeuroImage.

[47] Benjamini Y., Hochberg Y. (1995). Controlling the False Discovery Rate: A Practical and Powerful Approach to Multiple Testing. J. R. Stat. Soc. Ser. B Methodol..

[48] Hahamy A., Behrmann M., Malach R. (2015). The idiosyncratic brain: Distortion of spontaneous connectivity patterns in autism spectrum disorder. Nat. Neurosci..

[49] Thye M.D., Bednarz H.M., Herringshaw A.J., Sartin E.B., Kana R.K. (2018). The impact of atypical sensory processing on social impairments in autism spectrum disorder. Dev. Cogn. Neurosci..

[50] Pegado F., Hendriks M.H.A., Amelynck S., Daniels N., Steyaert J., Boets B., Op de Beeck H. (2020). Adults with high functioning autism display idiosyncratic behavioral patterns, neural representations and connectivity of the ‘Voice Area’ while judging the appropriateness of emotional vocal reactions. Cortex.

[51] Safar K., Wong S.M., Leung R.C., Dunkley B.T., Taylor M.J. (2018). Increased Functional Connectivity During Emotional Face Processing in Children With Autism Spectrum Disorder. Front. Hum. Neurosci..

[52] Cerliani L., Mennes M., Thomas R.M., Di Martino A., Thioux M., Keysers C. (2015). Increased Functional Connectivity Between Subcortical and Cortical Resting-State Networks in Autism Spectrum Disorder. JAMA Psychiatry.

[53] Khan A.J., Nair A., Keown C.L., Datko M.C., Lincoln A.J., Müller R.-A. (2015). Cerebro-cerebellar Resting-State Functional Connectivity in Children and Adolescents with Autism Spectrum Disorder. Biol. Psychiatry.

[54] Eyler L.T., Pierce K., Courchesne E. (2012). A failure of left temporal cortex to specialize for language is an early emerging and fundamental property of autism. Brain.

[55] Herbert M.R., Ziegler D.A., Deutsch C.K., O’Brien L.M., Kennedy D.N., Filipek P.A., Bakardjiev A.I., Hodgson J., Takeoka M., Makris N. (2005). Brain asymmetries in autism and developmental language disorder: A nested whole-brain analysis. Brain.

[56] Herringshaw A.J., Ammons C.J., DeRamus T.P., Kana R.K. (2016). Hemispheric differences in language processing in autism spectrum disorders: A meta-analysis of neuroimaging studies. Autism Res..

[57] Jouravlev O., Kell A.J., Mineroff Z., Haskins A.J., Ayyash D., Kanwisher N., Fedorenko E. (2020). Reduced Language Lateralization in Autism and the Broader Autism Phenotype as Assessed with Robust Individual-Subjects Analyses. Autism Res..

[58] Kleinhans N.M., Müller R.-A., Cohen D.N., Courchesne E. (2008). Atypical functional lateralization of language in autism spectrum disorders. Brain Res..

[59] Lindell A.K., Hudry K. (2013). Atypicalities in Cortical Structure, Handedness, and Functional Lateralization for Language in Autism Spectrum Disorders. Neuropsychol. Rev..

[60] Knaus T.A., Silver A.M., Lindgren K.A., Hadjikhani N., Tager-Flusberg H. (2008). fMRI activation during a language task in adolescents with ASD. J. Int. Neuropsychol. Soc..

[61] Asaridou S.S., Demir-Lira E., Udden J.S., Goldin-Meadow S., Small S.L. (2019). Pragmatic Language Processing in the Adolescent Brain. BioRxiv.

[62] Pina-Camacho L., Villero S., Fraguas D., Boada L., Janssen J., Navas-Sánchez F.J., Mayoral M., Llorente C., Arango C., Parellada M. (2012). Autism Spectrum Disorder: Does Neuroimaging Support the DSM-5 Proposal for a Symptom Dyad? A Systematic Review of Functional Magnetic Resonance Imaging and Diffusion Tensor Imaging Studies. J. Autism Dev. Disord..

[63] Schumann C. (2021). Limbic System. Encyclopedia of Autism Spectrum Disorders.

[64] Catani M., Dell’acqua F., de Schotten M.T. (2013). A revised limbic system model for memory, emotion and behaviour. Neurosci. Biobehav. Rev..

[65] Besseling R., Lamerichs R., Michels B., Heunis S., de Louw A., Tijhuis A., Bergmans J., Aldenkamp B. (2018). Functional network abnormalities consistent with behavioral profile in Autism Spectrum Disorder. Psychiatry Res. Neuroimaging.

[66] Reveley M.A., Deakin J.F.W., Reveley M.A., Deakin J.F.W., British Association for Psychopharmacology (1999). The Psychopharmacology of Schizophrenia.

[67] Larson C., Thomas H.R., Crutcher J., Stevens M.C., Eigsti I.M. (2023). Language Networks in Autism Spectrum Disorder: A systematic review of connectivity-based fMRI studies. Rev. J. Autism Dev. Disord..

[68] Zhou R., Sun C., Sun M., Ruan Y., Li W., Gao X. (2024). Altered intra- and inter-network connectivity in autism spectrum disorder. Aging.

[69] Chenji S., Jha S., Lee D., Brown M., Seres P., Mah D., Kalra S. (2016). Investigating Default Mode and Sensorimotor Network Connectivity in Amyotrophic Lateral Sclerosis. PLoS ONE.

[70] Marrus N., Eggebrecht A.T., Todorov A., Elison J.T., Wolff J.J., Cole L., Gao W., Pandey J., Shen M.D., Swanson M.R. (2018). Walking, Gross Motor Development, and Brain Functional Connectivity in Infants and Toddlers. Cereb. Cortex.

[71] Buckner R.L., Andrews-Hanna E.J.R., Schactera D.L. (2008). The Brain’s Default Network: Anatomy, Function, and Relevance to Disease. Ann. N. Y. Acad. Sci..

[72] Raichle M.E., MacLeod A.M., Snyder A.Z., Powers W.J., Gusnard D.A., Shulman G.L. (2001). A Default Mode of Brain Function. Proc. Natl. Acad. Sci. USA.

[73] Chen H., Duan X., Liu F., Lu F., Ma X., Zhang Y., Uddin L.Q., Chen H. (2016). Multivariate classification of autism spectrum disorder using frequency-specific resting-state functional connectivity—A multi-center study. Prog. Neuro-Psychopharmacol. Biol. Psychiatry.

[74] Glerean E., Pan R.K., Salmi J., Kujala R., Lahnakoski J.M., Roine U., Nummenmaa L., Leppämäki S., Wendt T.N., Tani P. (2016). Reorganization of functionally connected brain subnetworks in high-functioning autism. Hum. Brain Mapp..

[75] Hull L., Petrides K.V., Allison C., Smith P., Baron-Cohen S., Lai M.-C., Mandy W. (2017). “Putting on My Best Normal”: Social Camouflaging in Adults with Autism Spectrum Conditions. J. Autism Dev. Disord..

[76] Lombardo M.V., Chakrabarti B., Bullmore E.T., Sadek S.A., Pasco G., Wheelwright S.J., Suckling J., Baron-Cohen S., MRC AIMS Consortium (2010). Atypical neural self-representation in autism. Brain.

[77] Lynch C.J., Uddin L.Q., Supekar K., Khouzam A., Phillips J., Menon V. (2013). Default Mode Network in Childhood Autism: Posteromedial Cortex Heterogeneity and Relationship with Social Deficits. Biol. Psychiatry.

[78] Morita T., Kosaka H., Saito D.N., Ishitobi M., Munesue T., Itakura S., Omori M., Okazawa H., Wada Y., Sadato N. (2012). Emotional responses associated with self-face processing in individuals with autism spectrum disorders: An fMRI study. Soc. Neurosci..

[79] Murdaugh D.L., Nadendla K.D., Kana R.K. (2014). Differential role of temporoparietal junction and medial prefrontal cortex in causal inference in autism: An independent component analysis. Neurosci. Lett..

[80] Nielsen J.A., Zielinski B.A., Fletcher P.T., Alexander A.L., Lange N., Bigler E.D., Lainhart J.E., Anderson J.S. (2013). Multisite functional connectivity MRI classification of autism: ABIDE results. Front. Hum. Neurosci..

[81] Pantelis P.C., Byrge L., Tyszka J.M., Adolphs R., Kennedy D.P. (2015). A specific hypoactivation of right temporo-parietal junction/posterior superior temporal sulcus in response to socially awkward situations in autism. Soc. Cogn. Affect. Neurosci..

[82] Yerys B.E., Gordon E.M., Abrams D.N., Satterthwaite T.D., Weinblatt R., Jankowski K.F., Strang J., Kenworthy L., Gaillard W.D., Vaidya C.J. (2015). Default mode network segregation and social deficits in autism spectrum disorder: Evidence from non-medicated children. NeuroImage Clin..

[83] Ypma R.J., Moseley R.L., Holt R.J., Rughooputh N., Floris D.L., Chura L.R., Spencer M.D., Baron-Cohen S., Suckling J., Bullmore E.T. (2016). Default Mode Hypoconnectivity Underlies a Sex-Related Autism Spectrum. Biol. Psychiatry Cogn. Neurosci. Neuroimaging.

[84] Eggebrecht A.T., Elison J.T., Feczko E., Todorov A., Wolff J.J., Kandala S., Adams C.M., Snyder A.Z., Lewis J.D., Estes A.M. (2017). Joint Attention and Brain Functional Connectivity in Infants and Toddlers. Cereb. Cortex.

[85] McKinnon C.J., Eggebrecht A.T., Todorov A., Wolff J.J., Elison J.T., Adams C.M., Snyder A.Z., Estes A.M., Zwaigenbaum L., Botteron K.N. (2019). Restricted and Repetitive Behavior and Brain Functional Connectivity in Infants at Risk for Developing Autism Spectrum Disorder. Biol. Psychiatry Cogn. Neurosci. Neuroimaging.

[86] Wang Q., Li H.-Y., Li Y.-D., Lv Y.-T., Ma H.-B., Xiang A.-F., Jia X.-Z., Liu D.-Q. (2021). Resting-state abnormalities in functional connectivity of the default mode network in autism spectrum disorder: A meta-analysis. Brain Imaging Behav..

[87] Vissers M.E., Cohen M.X., Geurts H.M. (2012). Brain connectivity and high functioning autism: A promising path of research that needs refined models, methodological convergence, and stronger behavioral links. Neurosci. Biobehav. Rev..

[88] Menon V., Uddin L.Q. (2010). Saliency, switching, attention and control: A network model of insula function. Brain Struct. Funct..

[89] Fox M.D., Corbetta M., Snyder A.Z., Vincent J.L., Raichle M.E. (2006). Spontaneous Neuronal Activity Distinguishes Human Dorsal and Ventral Attention Systems. Proc. Natl. Acad. Sci. USA.

[90] Elton A., Di Martino A., Hazlett H.C., Gao W. (2016). Neural Connectivity Evidence for a Categorical-Dimensional Hybrid Model of Autism Spectrum Disorder. Biol. Psychiatry.

[91] Farrant K., Uddin L.Q. (2016). Atypical developmental of dorsal and ventral attention networks in autism. Dev. Sci..

[92] Uddin L.Q., Yeo B.T.T., Spreng R.N. (2019). Towards a Universal Taxonomy of Macro-scale Functional Human Brain Networks. Brain Topogr..

[93] Yerys B.E., Herrington J.D., Satterthwaite T.D., Guy L., Schultz R.T., Bassett D.S. (2017). Globally weaker and topologically different: Resting-state connectivity in youth with autism. Mol. Autism.

[94] Lee P.S., Yerys B.E., Della Rosa A., Foss-Feig J., Barnes K.A., James J.D., VanMeter J., Vaidya C.J., Gaillard W.D., Kenworthy L.E. (2008). Functional Connectivity of the Inferior Frontal Cortex Changes with Age in Children with Autism Spectrum Disorders: A fcMRI Study of Response Inhibition. Cereb. Cortex.

[95] Odriozola P., Uddin L.Q., Lynch C.J., Kochalka J., Chen T., Menon V. (2016). Insula response and connectivity during social and non-social attention in children with autism. Soc. Cogn. Affect. Neurosci..

[96] Solomon M., Ozonoff S.J., Ursu S., Ravizza S., Cummings N., Ly S., Carter C.S. (2009). The neural substrates of cognitive control deficits in autism spectrum disorders. Neuropsychologia.

[97] Cakar M.E., Okada N.J., Cummings K.K., Jung J., Bookheimer S.Y., Dapretto M., Green S.A. (2024). Functional connectivity of the sensorimotor cerebellum in autism: Associations with sensory over-responsivity. Front. Psychiatry.

[98] Wang S.H., Kloth A.D., Badura A. (2014). The Cerebellum, Sensitive Periods, and Autism. Neuron Perspective..

[99] Clark S.V., King T.Z., Turner J.A. (2020). Inhibitory control in the cerebellum is facilitated by internal models. J. Psychopathol. Behav. Assess..

[100] Zarka D., Cebolla A.M., Cheron G. (2022). Mirror neurons, neural substrate of action understanding? A review. Neurosci. Biobehav. Rev..

[101] Wahl M.L., Serra I., Badura A. (2024). Impact of cerebellar-specific genetic and circuit manipulations on the behavioral phenotype and cerebellar physiology in murine autism models. Brain Res..

[102] Guerra A.B., De Meo C.R., Oliveira G.A. (2024). Unraveling the Cerebellar Involvement in Autism Spectrum Disorders: Insights into Genetic Mechanisms and Developmental Pathways. Cells.

[103] Kong P., Chen Y., Song W. (2024). Cerebellum and social abilities: A structural and functional connectivity study in a transdiagnostic sample. Hum. Brain Mapp..

[104] Sivalingam A.M., Pandian A. (2024). Cerebellar Roles in Motor and Social Functions and Implications for ASD. Cerebellum.

